# Development, explanation, and presentation of the Physical Literacy Interventions Reporting Template (PLIRT)

**DOI:** 10.1186/s12966-023-01423-3

**Published:** 2023-02-18

**Authors:** Johannes Carl, Jaime Barratt, Kelly P. Arbour-Nicitopoulos, Lisa M. Barnett, Dean A. Dudley, Peter Holler, Richard Keegan, Matthew Kwan, Raffaele Scurati, Raymond Kim-Wai Sum, Nalda Wainwright, John Cairney

**Affiliations:** 1 Department of Sport Science and Sport, Friedrich-Alexander University Erlangen-Nürnberg, Gebbertstraße 123b, 91058 Erlangen, Germany; 2 School of Human Movement and Nutrition Sciences, The University of Queensland, Qld 4072 Brisbane, Australia; 3 Faculty of Kinesiology and Physical Education, University of Toronto, 55 Harbord Street, ON Toronto, Canada; 4 Faculty of Health, Institute for Physical Activity and Nutrition, School of Health and Social Development, Deakin University, 221 Burwood Hwy, Burwood, 3147 Melbourne, Australia; 5 Macquarie School of Education, Macquarie University, 1 University Ave, 2109 Sydney, Australia; 6 School of Human Movement and Nutrition Sciences, The University of Queensland, Qld 4072 Brisbane, Australia; 7 FH JOANNEUM, Institute of Health and Tourism Management, Kaiser-Franz-Josef-Straße 24, 8344 Bad Gleichenberg, Austria; 8 Faculty of Health Canberra, Research Institute for Sport and Exercise (UC-RISE), University of Canberra, ACT 2617 Canberra, Australia; 9 Faculty of Social Sciences, Brock University, Child and Youth Studies, 1812 Sir Isaac Brock Way , ON St. Catharines, Canada; 10 Department of Biomedical Sciences for Health, University of Milan, Via Kramer 4/4A, 20129 Milano, Italy; 11 Department of Sports Science and Physical Education, Faculty of Education, The Chinese University of Hong Kong, G09, Kwok Sports Building, Hong Kong, Hong Kong SAR; 12 Wales Academy for Health and Physical Literacy, University of Wales Trinity Saint David , College Road, Wales SA31 3EP Carmarthen, Great Britain

**Keywords:** Physical activity, Health, Theory, Physical Education, Study Quality

## Abstract

**Background:**

The physical literacy (PL) concept integrates different personal (e.g., physical, cognitive, psychological/affective, social) determinants of physical activity and has received growing attention recently. Although practical efforts increasingly adopt PL as a guiding concept, latest evidence has shown that PL interventions often lack specification of important theoretical foundations and basic delivery information. Therefore, the goal of the present study was to develop an expert-based template that supports researchers and practitioners in planning and reporting PL interventions.

**Methods:**

The development process was informed by Moher et al.’s guidance for the development of research reporting guidelines. We composed a group of ten distinguished experts on PL. In two face-to-face meetings, the group first discussed a literature-driven draft of reporting items. In the second stage, the experts anonymously voted and commented on the items in two rounds (each leading to revisions) until consensus was reached.

**Results:**

The panel recommended that stakeholders of PL initiatives should tightly interlock interventional aspects with PL theory while ensuring consistency throughout all stages of intervention development. The Physical Literacy Interventions Reporting Template (PLIRT) encompasses a total of 14 items (two additional items for mixed-methods studies) in six different sections: title (one item), background and definition (three items), assessment (one item each for quantitative and qualitative studies), design and content (five items), evaluation (one item plus one item each for quantitative and qualitative studies), discussion and conclusion (two items).

**Conclusion:**

The PLIRT was designed to facilitate improved transparency and interpretability in reports on PL interventions. The template has the potential to close gaps between theory and practice, thereby contributing to more holistic interventions for the fields of physical education, sport, and health.

**Supplementary Information:**

The online version contains supplementary material available at 10.1186/s12966-023-01423-3.

## Background

### Physical literacy

Bolstered by increasing evidence that physical activity exerts positive influences on physical health [1, 2], psychological well-being [3], and academic success [4], both researchers and practitioners search for concepts that support individuals in their long-term adoption of a physically active lifestyle. When comprehending such an adoption as a lifelong individual pursuit, the term *literacy* becomes highly relevant and appropriate, due to its focus on long-term learning processes [5]. Indeed, having its roots in the late 19^th^ and early twentieth century [6], recent research on physical activity, health, and physical education has demonstrated growing interest in physical literacy (PL) as a holistic concept for the specific area of physical activities [7, 8]. According to the International Physical Literacy Association (IPLA), PL can be understood as “the motivation, confidence, physical competence, knowledge, and, understanding to value and take responsibility for engagement in physical activities for life” [9].

Although there has been an exponential increase in academic contributions to PL, the research field is currently far away from achieving a shared consensus regarding this subject, which is mirrored by a large number of recent articles discussing PL from a conceptual perspective [7, 8, 10–12]. For instance, the Australian framework emphasizes the relevance of a social component for PL [13, 14], while New Zealand added a spiritual element to the discussion [15]. Young et al. scientifically mapped the PL landscape with respect to main actors and identified four clusters of researchers cultivating different perspectives of PL [16]. Despite the plurality of concepts and approaches, which can from a theory of science perspective be considered as rather typical for a dynamic research field [17], the IPLA conceptualization can be described as representing the smallest intersection of postulated components among the different clusters. In line with this definition, PL can be characterized through several domains: an affective domain (“motivation and confidence”), a physical domain (“physical competence”), a cognitive domain (“knowledge and understanding”), and a behavioral domain (“physical activities for life”) [18, 19]. Importantly, these domains do not stand in isolation from one other. Instead, the domains show reciprocal reinforcements [20–22] and are strongly intertwined [18], which uncouples the descriptions from a dualistic worldview and harmonizes with monistic and embodied positions [23]. In general, PL has already undergone extensive discussions regarding its philosophic tenets and has found further roots in existentialism and phenomenology [8, 11, 23, 24]. Whitehead, as one of the main advocates of PL in the early stages, repeatedly stressed that it is hardly possible to comprehend PL without embracing its philosophical foundations [12]. As one of the central assumptions of PL, phenomenology holds that all experiences build on individual biographies and, therefore, provide “unique journeys” in interaction with the environment [25, 26]. Accordingly, learning is an ongoing process with PL warranting relevance throughout the whole lifecourse [27]. The person-centered considerations, in turn, open the concept for all individuals. The openness not only refers to healthy individuals of any age [28, 29] but also to people with developmental disorders or disabilities as previous overview articles have underlined the particular value of PL for inclusive efforts [30, 31].

### Physical literacy interventions

PL can be fostered systematically through structured experiences, such as education, training, coaching, or clinical applications [32, 33]. This, in turn, places more of a responsibility on the providers of such training, such as teachers [34], educators [35], coaches [36], social workers [37], and therapists [38]. Accordingly, and mirroring developments in most areas of science, researchers have increasingly attempted to translate the theoretical tenets of PL into practical interventions. Of course, such a translation from theoretical ideas and controlled tests into the “real world” often requires a degree of compromise and pragmatism [39]. Interventional initiatives have been set up with the target groups of preschoolers [35], school age children [40], adolescents [41], university students [42], and adults [43]. Interventions also span across different settings such as schools [44], sport clubs [36], community facilities [45], as well as hospitals [38]. Indeed, reviewing the current literature has suggests multiple different approaches with respect to PL interventions. Nevertheless, a recent systematic review on the design and content of PL interventions has highlighted that, overall, the conceptual tenets underlying PL insufficiently imbue the interventional techniques [46]. In particular, that review demonstrated that physical competence has been addressed in 84%, but knowledge and understanding only in 59% and motivation and confidence only in 48% of all intervention studies [46]. Specifically, Kwan et al. [42] pointed out that “PL-based programs must be developed with intentionality that is aimed at all of its core domains” (p. 2). However, the review showed that existing PL interventions do not deliver on this intent [46], irrespective of the recommendation that behavior-related interventions are advised to cultivate explicit theory-content links within their reports [47]. Although this overall picture is in line with conceptual analyses revealing a disproportionate emphasis on the physical domain [10, 48], this finding raises doubts as to whether current practices truly account for the embodied and integrated nature of the PL concept. In addition, the studies included in that review often lack basic technical information including intervention length, duration, frequency, and intensity [46]. Therefore, better harmonization of the research field with general quality-ensuring guidelines such as the template for intervention description and replication (TIDieR) checklist [49] or the consensus on exercise reporting template (CERT) [50] would be indicated. Against this background, the PL literature could benefit from explicit standards on how to design and report interventions that declare to be based on the concept; both regarding the planning and reporting of interventions, but also regarding the approach taken to PL itself.

### Goal of the article

In response to the above-identified needs, the goal of the present study was to develop a specific reporting template that supports researchers and practitioners planning, reporting, and interpreting PL intervention research, serving as a minimally expected guiding framework for the reporting of study results. The present initiative aims at improving the infusion of important theoretical aspects into intervention studies and, hence, at enhancing the quality of the interventional landscape regarding PL. More specifically, the reporting template was developed by different experts and provides the potential starting point for studies exploring the value of the guideline from an empirical perspective. In the long term, this endeavor seeks to promote the holistic health of individuals and societies, as interventions may be more successful in simultaneously integrating and explicitly addressing the physical, affective, cognitive, and behavioral determinants of physical activity [22]. Accordingly, our study was guided by the following research question: “What are the minimum criteria that experts/researchers need to report, in order to appropriately understand, interpret, and synthesize PL interventions?”.

## Methods

### The development process

**Fig. 1:**
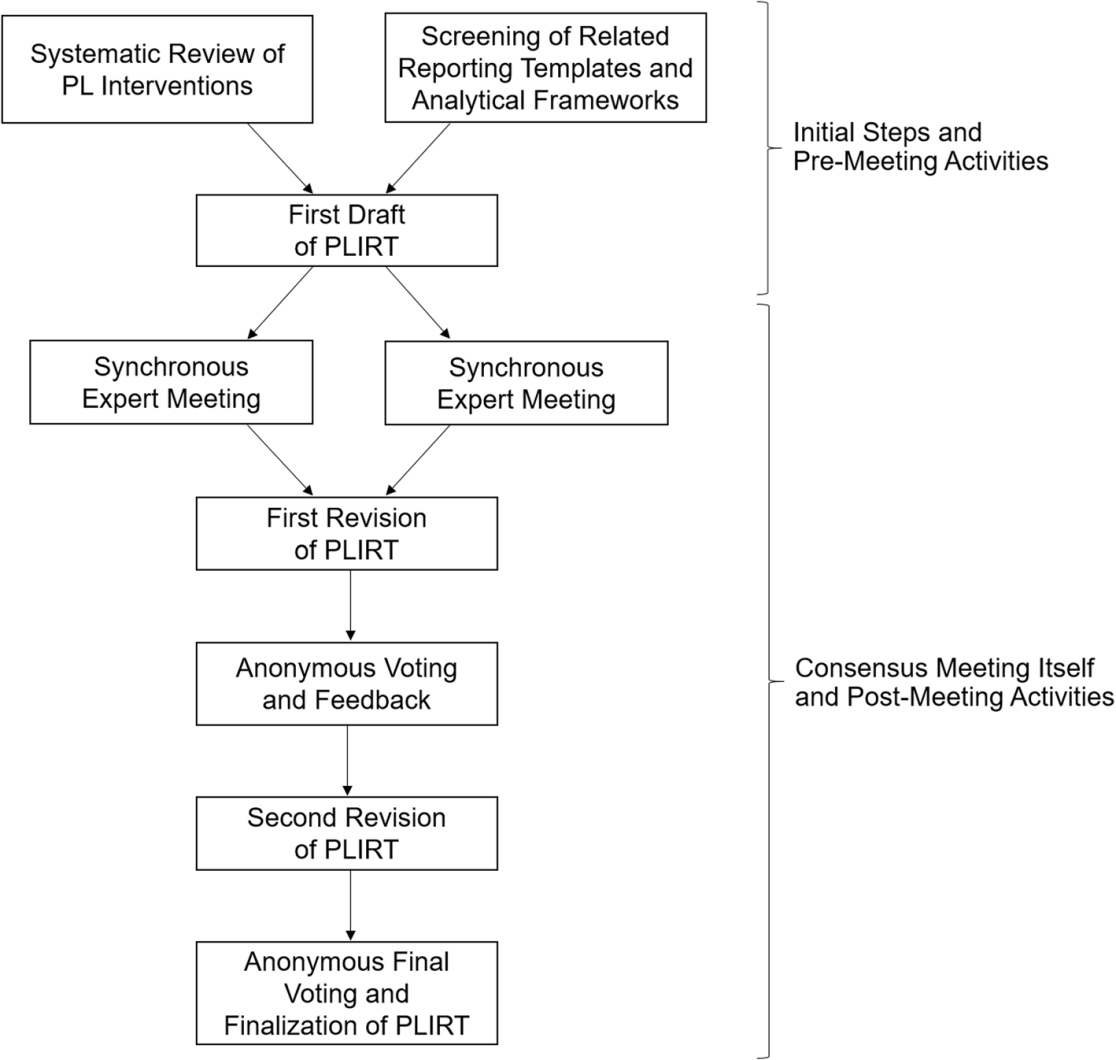
Methodological steps for the development of the Physical Literacy Interventions Reporting Template (PLIRT)

The development of the “Physical Literacy Interventions Reporting Template” (PLIRT) was informed by Moher et al.’s guidance for the development of research reporting guidelines [51]. We chose an expert-based consensus approach to facilitate diversity in perspectives, and thus seeking a final product that accommodates and supports these different approaches. Specific to this initiative, a consensus method harmonized well with the purpose due to following “the call to improve practice by capitalizing on accumulated practical experience and using this to develop better interventions” [52]. In general, consensus methods have to be thoroughly aligned with the current state of the literature, the specificities of a scientific approach, and the purpose of a study project. Accordingly, the suggested procedure can undergo modifications, if necessary [53, 54]. Nevertheless, the development process (see Fig. 1) deviated only in two points from the suggested guidance [51]. First, the Delphi exercise was split into two face-to-face meetings with two subsequent rounds of anonymous feedback and voting. Second, in line with these extensions in the post-meeting phase, we considered the pilot-test of the reporting template as part of a potential, more empirically focused publication after this initial suggestion (see also publication strategy in item 11). All items of the aforementioned guidance are commented in supplementary Table 1.

### Initial steps and pre-meeting activities

In line with recommendations regarding the adequate number of experts in consensus projects [55], we invited a total of eleven experts to join a discussion for reporting requirements of PL interventions. To be considered as experts, the invited persons must have published at least three studies on PL or conducted at least one PL intervention as a first/senior author. To cover a broad spectrum of heterogeneous experts, the following selection criteria were considered: (a) network background – the experts should represent different clusters of researchers as found by Young et al. [16] (i.e., the “idealist embodiment cluster”, the “idealist-pragmatic cluster”, the “pragmatic health determinant cluster”, and the “pragmatic disease prevention cluster”) to not disproportionately promote a specific and selected perspective of PL; (b) geography – the experts had to adequately cover the continental areas where PL interventions were dominantly conducted in the past (i.e., North America, Europe, Asia, and Australia) [46]; (c) gender – the expert group should have been composed by a diversity of gender; and (d) age – the group should comprise researchers at different stages of career. Nine of the eleven contacted experts directly accepted the invitation and completed the whole process (81.8%). One expert withdrew the commitment before the first meeting, while another declined the offer due to a lack of time but suggested a colleague who fit the expert criteria and joined the project. Based on the identification of common issues in PL interventions (gained through a systematic review [46]) and an initial screening of related frameworks, the first author of the current study worked out an initial pool of potential PLIRT items, which were sent out to all participating experts prior to the first meeting.

### Face-to-face meeting and post-meeting activities

Due to considerable time zone differences among expert group members, we facilitated synchronous discussion in two separate meetings (North America and Europe; Asia and Australia). Both (approximately 90 min in duration) were held online via zoom v5.10 (Zoom Video Communications, San José, United States) and lasted approximately 90 min in duration. The meeting started with an introduction of the participating experts as well as a presentation of the results of the systematic review on PL interventions, highlighting again the need for improving the reporting quality among PL interventions [46]. Afterwards, the expert group critically discussed the initially suggested items. The first author of the current study moderated both meetings. With the help of meeting protocols and a video recording, the core team (JC, JB) subsequently revised the PLIRT draft at 15 positions (across 11 of the 14 items). Corresponding to an iterative process, all participating experts were subsequently invited to evaluate all revised PLIRT items individually and anonymously via an online survey (three rating options: completely agree with the item and description; agree with the item and the description but suggest a modification; disagreement with the items and the description). In cases of not fully agreeing with an item and its description, the experts had the opportunity to comment on the item and suggest a revision. Items were considered as “finalized” if (a) at least 80% of all experts completely agreed with this item without any modification suggestions, and (b) if there was simultaneously not more than one single expert disagreeing with this item. After the first round of feedback, the core team performed a revision of the reporting template (across 5 of the 14 item formulations and across 12 of the 14 item descriptions) as the results and comments of the survey revealed that the finalization criterion was not given for all items. After the second round of individual, anonymous feedback and voting with the same rating options, the results revealed sufficient agreement to the PLIRT. The only exception was item #6 with an agreement rate of 70%. Inspection of the comments showed that the experts only suggested linguistic (and not thematic) revisions for this item. In consideration of this, the core team decided to directly address the comments related to this item while not handing the PLIRT back to all participating experts for a third time. Once completed, the finalized status of the reporting template was acknowledged. The agreement statistics of the two feedback and revision rounds can be retrieved from Table 1.

**Table 1:** Results of the voting and feedback

Item	First Round of Anonymous Feedback and Voting			Second Round of Anonymous Feedback and Voting		
	Complete Agreement	Agreement but Modification Suggestion	Disagreement	Complete Agreement	Agreement but Modification Suggestion	Disagreement
**1**	8 (80%)	2 (20%)	0	10 (100%)	0	0
**2**	6 (60%)	4 (40%)	0	10 (100%)	0	0
**3**	3 (30%)	7 (70%)	0	9 (90%)	1 (10%)	0
**4**	8 (80%)	2 (20%)	0	10 (100%)	0	0
**5a**	7 (70%)	3 (30%)	0	9 (90%)	1 (10%)	0
**5b**	6 (60%)	4 (40%)	0	9 (90%)	1 (10%)	0
**6**	4 (40%)	5 (50%)	1 (10%)	7 (70%)	3 (30%)	0
**7**	8 (80%)	1 (10%)	1 (10%)	9 (90%)	1 (10%)	0
**8**	8 (80%)	2 (20%)	0	9 (90%)	1 (10%)	0
**9**	6 (60%)	3 (30%)	1 (10%)	8 (80%)	1 (10%)	1 (10%)
**10**	10 (100%)	0	0	-		
**11**	7 (70%)	3 (30%)	0	8 (80%)	2 (20%)	0
**12a**	10 (100%)	0	0	-		
**12b**	7 (70%)	3 (30%)	0	10 (100%)	0	0
**13**	7 (70%)	2 (20%)	1 (10%)	9 (90%)	1 (10%)	0
**14**	8 (80%)	2 (20%)	0	10 (100%)	0	0

Note: The table cells report the absolute numbers outside the brackets and the relative numbers (in percentage) inside the brackets; see Fig. 1 for a visualization of the whole process

### How to use this paper

We present each item with a separate heading in the results section and awarded a separate line within an overview table. Short and concise explanations are given for each item, between 100 and 250 words in length. We strongly encourage authors of PL interventions to include the information of the 14 (or 16, respectively) items into their study reports. Even though the items here are listed in numerical order from 1 to 14, authors need not to address the items in their reports in this particular sequence. Instead, we recommend that the PLIRT table should be outlined at least in the supplemental material of a study while pointing to the location in the manuscript where the corresponding information is found (e.g., similar to the PRISMA statement guidelines for systematic reviews [56]). Most importantly, the PLIRT was designed to meet only the specificities of the PL concept. Accordingly, the present template should be used in conjunction with suggestions of other intervention guidelines, such as the TIDieR [49] checklist or CERT [50]. Against this background, an item (item #10) was provided to specifically underline this function. In general, the template only serves as a minimum standard for the reporting, and authors may choose to pursue further clarity, transparency, or rigor than is detailed here. In recognition of the differences in study types and general circumstances across PL interventions, authors should enclose further information if considered necessary.

## Results

### Overview

A total of 14 items were ultimately considered to adequately represent those aspects which should be covered by reports and studies on PL interventions (see Table 2). The 14 items referred to six sections: title, background and definition, assessment, design and content, evaluation, discussion and conclusion. For two items, the experts differentiated between quantitative (Items #5a, #12a) and qualitative PL endeavors (Items #5b, #12b). All items are listed and explained in more detail below.

**Table 2:** The PLIRT checklist

Item Nr.	Description	Location in the Paper and/or Comment
**TITLE**		
1	Highlight the role of PL in the title	
**BACKGROUND AND DEFINITION**		
2	Describe the relevance of PL for the target population/group/individual	
3	Explain your conceptualization of PL and refer to a holistic definition of PL	
4	Formulate PL-related goals/aims of your study	
**ASSESSMENT**		
5a	*If quantitative:* Choose a multidimensional assessment strategy of PL and provide information about psychometric properties	
5b	*If qualitative:* Develop a qualitative method that closely aligns with PL theory and the different domains	
**DESIGN AND CONTENT**		
6	Ensure that your interventional approach is in line with PL-compatible philosophical assumptions	
7	Mention the intervention provider(s), describe his/her/their expertise specific to PL, and any specific training given	
8	Report in detail intervention content related to all PL domains	
9	Explain whether and how you realized the integrative arrangement of content/techniques	
10	Consider general guidelines for intervention reporting	
**EVALUATION**		
11	Describe how the PL intervention was accepted and/or whether it was implemented as intended (modifications, fidelity, compliance, adherence)	
12a	*If quantitative:* Report transparently how the different PL domains (and, if initially intended, other relevant outcomes such as health) were affected by the intervention	
12b	*If qualitative:* Characterize the strengths, weaknesses, and challenges of your PL intervention; the different PL domains may help you structure the analysis and results	
**DISCUSSION AND CONCLUSION**		
13	Discuss the limitations of your PL intervention, especially whether (if yes, where and why) you had to deviate meaningfully from your planned conceptualization	
14	Break down your experiences with the PL intervention and derive sound recommendations for future studies	

Note: *PLIRT* Physical Literacy Interventions Reporting Template, *PL* Physical Literacy

### Item 1: Title – highlight the role of PL in the title

The title of an article often attracts initial attention of readers. By summarizing the primary focus of an article with a limited number of words and signs, a concise title often helps readers making the decision of whether to read or not read the full study. Explicitly outlining PL as a concept gives the reader orientation regarding the broad theoretical lens adopted by a study. In addition, mentioning the concept in title facilitates the inclusion of the study into literature reviews, such as systematic reviews or meta-analyses [49, 56]. Ideally, authors should deliberately use terms that adequately reflect the degree of theoretical foundation for their intervention [57]. For instance, formulations such as “PL-informed” or “PL-inspired” may characterize interventions with rather indirect, lose, or secondary theoretical foundations, while formulations such as “PL-driven”, “PL-based”, or simply “PL intervention” may highlight interventions with a dominant role of PL (for a similar suggestion, see [57, 58]). As a shorter alternative to explicitly mentioning PL in the title, authors may use the description “holistic” [59] or “multidimensional” for corresponding interventions.

### Item 2: Background and definition – describe the relevance of PL for the target population/group/individual

Interventions can span different levels, including whole populations, groups, or individuals. In this context, authors should be clear who the intervention is targeted to and make this information transparent. From a theoretical perspective, PL offers unique perspectives throughout the whole life course, which means that interventions can basically be developed for every individual [39, 46]. Regardless, by following targeted or tailored approaches, the design of interventions should be geared toward the needs of the target group/individual [60]. Therefore, the background section should stress the specific relevance of PL for the target group/individual. For instance, this can be realized by taking a developmental perspective that locates the study in a life journey [61]. This section should also describe if and why certain aspects or domains of PL were specifically emphasized in the intervention. In summary, this reporting item may help in deriving the intentionality of the intervention study.

### Item 3: Background and definition – explain your conceptualization of PL and refer to a holistic definition of PL

**Fig. 2:**
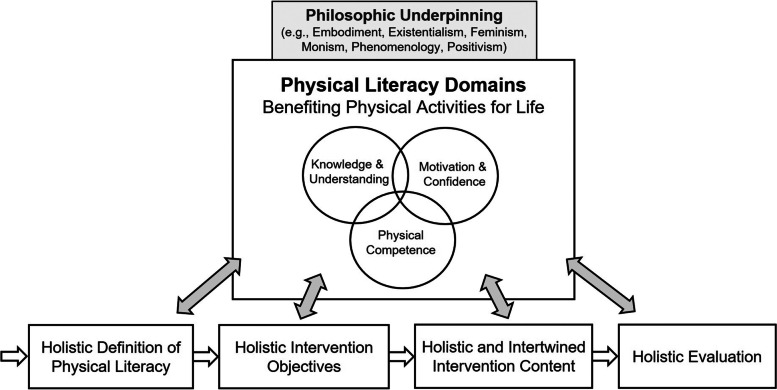
Cultivating the holistic character through a tight and consistent interlocking with the PL domains at all stages of the intervention process (revised figure [46]). Note: This figure visualizes the physical, cognitive, and affective domains as the minimum standard of PL domains; however, in accordance with the holistic framework selected, additional domains may come into play (e.g., a social domain in the Australian [13] or a spiritual element in the New Zealand framework [15])

The research field of PL contains different frameworks, definitions, models, and understandings of PL [8, 11, 12]. The goal of the present article is not to give recommendations regarding the “best” PL approach. Instead, the PLIRT acknowledges the pluralism of the field with its different perspectives about PL [10, 62]. Nevertheless, the PLIRT advocates for applying a holistic framework in ways that are aligned to the core meaning of the concept by acknowledging the embodied and integrated nature of human beings. This would mean that the selected conceptualization should encompass a definition that reflects at least physical, affective/psychological, and cognitive components in the context of physical activities as these can be found consistently across different countries and organizations (see Supplementary Table 2). As some examples, the definitions of the IPLA [9], in Canada [63], Australia [13], China [64], Wales [65], or New Zealand [15] all support such an approach. A growing number of definitions also specify social or spiritual aspects as constituting domains/elements of PL [13–15]. Specifically, this item aims at minimizing “uncouplings” from the’non-negotiable’ core meaning of PL [10]. Without this criterion being met, the intervention may ostensibly have diverged too far to be meaningfully understood as a “PL intervention”. A holistic understanding and definition represent the starting point of a consistent flow across the different stages of intervention reporting (Fig. 2).

### Item 4: Background and definition – formulate PL-related goals/aims of your study

In line with the initial description of the relevance of PL for the target group/individual and the definition selected, authors should derive explicit goals for their intervention. Ideally, researchers prospectively register their study or publish a study protocol with these goals [66, 67]. These goals may refer to PL as a holistic and inseparable concept or to the different domains. If researchers or practitioners decided to align their intervention goals with the different domains of PL, they may derive a multidimensional goal matrix [60]. Authors may also declare increases in PA levels (behavioral outcome) as worthwhile goals [35]. Given the postulated whole-person effects of PL, corresponding interventions may also intend to affect people’s general self-efficacy, overall resilience, health, or even quality of life [20–22, 68].

### Item 5a: Assessment (if quantitative) – choose a multidimensional assessment strategy of pl and provide information about psychometric properties

To meet the minimum criteria of a holistic approach, quantitative studies should choose a multidimensional assessment strategy that accounts for the multifaceted nature of PL by covering (at least) cognitive, physical, affective/psychological, and optionally behavioral, social, or spiritual aspects of physical activity. In line with Fig. 2 and item #3, the assessment should closely connect to the applied definition, with the PL domains potentially representing the decisive, common link between both stages. In this context, authors can reference review papers that have appraised assessment opportunities for PL [69–72] (recognizing that future reviews may become relevant to cite). Several established measurement instruments draw on different PL dimensions, such as the Canadian Assessment of Physical Literacy (CAPL-2), the Passport for Life (PFL), the Perceived Physical Literacy Inventory (PPLI), the Physical Literacy in Children Questionnaire (PL-C Quest), or the Physical Literacy Assessment for Youth (PLAY). It is typically recommended that, to show the specific fit of the assessment instrument(s) for the target group or population under interventional investigation, authors should provide information about the psychometric properties of their employed instrument(s) (e.g., reliabilities) [71]. Ideally, researchers transparently point to a more comprehensive validation study with the target group or population (e.g., indications of reliability, interrater agreement, content validity, construct/factorial validity, or criterion validity are presented) [73]. As a more economical alternative, researchers can describe the most important psychometric characteristics within the intervention study itself (e.g., in the “materials” of the methods section).

### Item 5b: Assessment (if qualitative) – develop a qualitative method that closely aligns with PL theory and the different domains

In accordance with the prominent philosophical roots of the concept, the field of PL interventions has yielded many qualitative approaches. When favoring a qualitative approach, authors should ensure that the methodological approach (e.g., interview guide, open-ended questionnaire format) reflects basic theoretical tenets of both the non-negotiable core of PL (holism, embodiment) and their own particular perspective (e.g., idealist, pragmatic, positivist) [39]. In this regard, each article can benefit from making the links between theory and the resulting method explicit. For instance, authors could comment how the selected philosophical stance (e.g., ideas of monism, phenomenology, and existentialism [23, 24]) aligned with methodological decisions. As another option, the methods may be deliberately constructed in accordance with the different domains of PL. In any way, the qualitative approach should be constructed in a way that an evaluation can adequately portray both positive and negative aspects of the intervention (i.e., through the avoidance of suggestive leading questions).

### Item 6: Design and content – ensure that your interventional approach is in line with PL-compatible philosophic assumptions

Given that “philosophy is the vital foundation behind physical literacy and that one cannot truly understand physical literacy without embracing its philosophical roots” [12], researchers should clearly explain the philosophical positioning of their study. In particular, authors should describe how their philosophical underpinnings permeated the interventional approach while avoiding that PL detaches too strongly from its original core [23]. Among the different philosophical underpinnings [23, 24], for instance, monistic assumptions call researchers and practitioners to comprehend body and mind as inseparable, interconnected units of human existence. Accordingly, when drawing on this philosophical assumption, it would be inappropriate to introduce isolated intervention blocs for each domain separately without a justification for how this will achieve the overall vision of PL, or to only apply physical stimuli for individuals (interventional neglection of the mind). On the grounds of existentialism, philosophic descriptions often highlight that individuals are formed by interactions with the environment. The reporting of PL interventions would be incomplete if no, or insufficient, information is given with respect to the social arrangement of the intervention and its embedded contexts, structures, settings, and environments. Furthermore, phenomenological reflections in the context of PL underline the uniqueness of individuals’ experiences. In this regard, interventions should avoid consequent one-size-fits-all solutions and *at least* integrate reflective phases that allow for these unique perspectives. In addition to these prominent philosophical underpinnings, researchers may also find crucial links to other philosophical positions that have not yet been elaborated in detail, such as positivism [39, 74], constructivism [75], or emancipatory feminism [76, 77]. In any case, researchers should ensure that the philosophical stance harmonizes with the interventional goals and content, and be transparent in how that harmonization is occurring.

### Item 7: Design and content – mention the intervention provider (s), describe his/her/their expertise specific to PL, and any specific training given

Studies should report the deliverer or provider of the intervention and state the topic-related expertise [49]. This item gains particular relevance in the PL context as differences in the effectiveness across different interventions may be attributed to differing understandings of the concept [78] and, therefore, to differing implicit intervention foci. Against this background, authors should clarify the disciplinary background (e.g., general pedagogy, physical education, psychology, licensed trainer, kinesiology, physiotherapy, exercise therapy, public health) and skills acquired. Furthermore, information regarding the amount of general expertise (e.g., number of years in the field or courses given) is helpful. Ultimately, if conducted, specific training (i.e., at least the focus and temporal volume) becomes relevant for study reporting.

### Item 8: Design and content – report in detail intervention content related to all PL domains

Questions regarding the’*what*’ and’*how*’ of the delivery constitute the core of an intervention. Authors should detail the contents and techniques that were applied in PL interventions. Moreover, the study should mention all materials that serve to support the application process (e.g., videos, drawings, brochures, training resources). In this context, we invite authors (and publishers) to draw on the opportunities of extensive supplemental material, if necessary, to ensure that readers can sufficiently comprehend what has been done. For greater clarification and comparability, authors may refer to standardized codings of behavior change techniques [79] or use observational tools for physical education practices [80]. Following the idea of intentionality, the holistic definition and goals of the previous phases should directly transform into contents and techniques to ensure a consistent conceptual flow throughout the intervention process. In this context, explicit links between theory (e.g., the different domains) and content may help to strengthen PL interventions’ conceptual base [46]. For instance, this could mean that each intended activity within a session (e.g., technical explanations, skill development, individual conditioning, cooperative games, reflection phases, peer feedback, autonomy supportive processes) is thoroughly connected to the underlying PL domain(s) (e.g., physical competence, social connectedness, knowledge, understanding, motivation, confidence) [81].

### Item 9: Design and content – explain whether and how you realized the integrative arrangement of content/techniques

One tenet of PL is the intertwining of physicality, cognition, and affect (see also the statements related to monism), which has already entailed reflections about consequences for practices [18]. Although this reporting requirement might be basically subsumed under the consideration of the philosophical background (see also item #6), we decided to provide a separate item for this interventional aspect. Authors should attempt to address all defined domains (which may also implicate a social or spiritual domain) in an integrative manner. Depending on the skills and the disciplinary background (see item #7), this may require close interprofessional collaboration between different experts [82, 83]. Accordingly, describe whether and how the intervention has provided such an integrative arrangement of content. A lack of consideration of this item should be identified as a limitation in the discussion section. For instance, researchers and practitioners could use methods of cognitive engagement [84] when performing physical activity (intertwining of physicality and cognition) under induction of an autonomy-supportive atmosphere (combination with an adequate motivational climate) to meet this claim [85]. Further examples of how to configure the intertwining in interventions can be found in a review about the design and content of PL interventions [46].

### Item 10: Design and content – consider general guidelines for intervention reporting

As explained in the *how to use this paper* section, we recommend authors to also consider guidelines of intervention reporting that are not related to PL. Depending on the focus of an intervention, the PLIRT could be combined with the TIDieR checklist [49] or the CERT [50]. For instance, sound reporting should comprise any information regarding intervention process [86] allowing the most trustworthy reproduction, not only length, duration, frequency, and intensity of the intervention. Further aspects may include the equipment, the format (individual vs. group based), supervision, specific tailoring, and the setting. If using a combination, reference should be made to both frameworks and guidelines. If no second framework or guideline is used, authors should explicitly mention this in the corresponding line for this item in the PLIRT table (e.g., in the supplemental material of an article). A recent systematic review from the health and disease context highlighted that studies insufficiently include the basic information of these two prominent reporting guidelines [87], which is why it is important to remind readers of the availability and use of such reporting standards.

### Item 11: Evaluation – describe how the PL intervention was accepted by the target individual/group and/or whether it was implemented as intended (modifications, fidelity, compliance, adherence)

The actual intervention may significantly deviate from the initially planned intervention. Despite the risk of affecting treatment integrity, modifications have the strong potential to facilitate the implementation and sustainability of interventions by improving the fit with the target individual/group or the embedded context [88]. Therefore, researchers should not hide such deviations but rather explain them transparently. Notably, modifications are not an exclusive phenomenon of PL literature [49, 89]. However, the PL concept is not always fully understood or accepted by providers [34]. Moreover, practitioners often prioritize the PL domains differently [90]. Against this background, the reporting should place particular attention on the involvement of information regarding treatment fidelity and modifications [88] as well as person compliance or adherence, respectively [91]. The responsible person(s) of an intervention should crucially document this information when preparing the planned program to ensure a proper registration of all activities performed. Ideally, this documentation is integrated into a more structured process evaluation that reflects important aspects of the intervention already during the implementation period (e.g., the subjective standpoint of relevant persons such as participants or deliverers) [86]. In summary, this information serves to generate explanations for the unexpected (non-)effectiveness of a PL intervention with the potential to improve the reach and/or effectiveness for upcoming endeavors. The limitations section constitutes an appropriate position where researchers can make reflections on the impact of such variations.

### Item 12a (if quantitative): Evaluation – report transparently how the different PL domains (and, if initially intended, other relevant outcomes such as health) were affected by the intervention

In line with the advocated consistent flow across the initial conceptualization and definition, the intervention goals and the intervention content and techniques, the article should report the results of all relevant outcomes. In addition to the results of the multidimensional instrument for assessing PL (see item #5a), authors are advised to also publish the results of the indicators that were argued to be additionally relevant for the intervention goals (see item #4). The concealment of evaluation outcomes represents a severe problem in intervention research due to its contribution to bias the value of the intervention approach under investigation (publication bias) [92].

### Item 12b (if qualitative): Evaluation – characterize the strengths, weaknesses, and challenges of your PL intervention; the different PL domains may help you structure the analysis and results

Attempt to present the results as rich, full, credible, and authentically as possible. This may include strengths, benefits, and facilitating mechanisms, on the one hand, but also weaknesses, challenges, and barriers of the intervention, on the other. The balance of its presentation should support the conclusions that are subsequently drawn from the study. The structure of the results section should adequately embody the theoretical focus adopted, the relevance of PL for the target individual/group, the goals of the intervention, and the initial construction strategy (no fundamental reprioritization of analytical aspects according to the valence of study results). In line with the holistic character of PL, a differentiation into the domains of the concept may help you structure the results section. In any case, the results should allow conclusions regarding the effectiveness of PL-related intervention components and/or the intertwining approach (see item #9).

### Item 13: Discussion and conclusion – discuss the limitations of your PL intervention, especially whether (if yes, where and why) you had to deviate meaningfully from your planned conceptualization

Authors are advised to disclose the limitations of their intervention study in the discussion section. There is some evidence that such information not only promotes transparency and scientific progress [93], but also that the disclosure of limitations does not substantially affect the reader’s confidence in the results and conclusions [94]. Typical limitations of PL interventions may include the absence of large sample sizes (especially when targeting persons with disabilities), lack of process evaluation, missing information about the intensity of activities performed, the need for specific validation studies for PL assessments as well as restrictions in generalizability due to the focus on a concrete, single setting. Unexpected and unavoidable “uncoupling” from original PL conceptualizations [10] in the course of an intervention gain particular relevance for the limitation section of interventions and should be discussed thoroughly. Specific to PL, limitations may also refer to an absence of the interventional intertwining of the PL domains (see item #9) or to incongruities between the philosophical stance and intervention content (see item #6). In summary, such a transparent reporting in the context of PL interventions can help to identify global challenges for improving the transfer of PL theory into practices and ideally daily routines.

### Item 14: Discussion and conclusion – Break down your experiences with the intervention and derive sound recommendations for future PL interventions

Given the insufficient translation of the conceptual ideas of PL into interventional endeavors, the PL field will strongly benefit from the experiences of single interventions. Accordingly, authors are advised to condense their *evidence-based experiences* into clear and concise main messages. Ideally, these messages take the form of recommendations with clear instructions for future PL intervention (e.g., structure, material, integrated intervention components). In the long term, the adoption of these recommendation may permeate daily practices to achieve a bigger public impact, from high quality in the physical education sector (e.g., early childhood, school age) to effective health promotion (e.g., people of older age and/or chronic diseases). In particular, there is a strong need for PL-related suggestions in older adults [46, 95], indigenous people and ethnic minorities [96, 97], persons with disabilities [31], and the general health context [98]. Depending on the results of the evaluation, PL studies may explicate the message to examine the intervention in new settings or with additional target groups and to systematically test this transfer (horizontal scale-up) [99]. In case of successful projects, authors can also explicitly communicate whether they consider the interventional evidence as eligible for a wider public impact by linking the intervention to higher organizational, geographical, or political spheres (vertical scale-up) [100, 101].

## Discussion

Researchers and practitioners increasingly draw on PL as a holistic concept to familiarize individuals, groups, or populations with different forms of physical activity. After conceptual discussions have dominated the research field of PL, a growing number of scholarly activities have recently dedicated their interest to the more pragmatic side of PL by developing interventional efforts on this concept. However, a review study has shown that the theoretical ideas of this person-centered approach have insufficiently permeated the design and content of these interventions [46]. Therefore, initiatives are required that improve the translation process between theory and the practices of physical education, sport, and health promotion [72]. The present article relies on the assumption that a reporting template—called PLIRT—has the potential to promote this translation process by suggesting items for the publication of intervention studies. It can be posited that a reporting template achieved through a formal expert consensus process, especially when used a priori, inspires stakeholders to consider aspects that may have been neglected without this endeavor. In this context, the present study has revealed that researchers have to integrate a minimum of 14 and 16 criteria across different sections of a research article, respectively, in order to appropriately understand, interpret, and synthesize PL interventions.

Future research should accumulate empirical data on the usefulness of the PLIRT (for further “post-publication activities”, see Supplementary Table 2). For instance, scholars could conduct interviews with future authors of PL interventions to directly acquire qualitative feedback on the PLIRT. As an alternative, researchers could compare the reporting quality through an external evaluation before and after the publication of PLIRT, or between upcoming PL intervention studies that explicitly mentioned the PLIRT versus not. Despite the need for empirical evidence, the present study used a systematic approach with experts of different perspectives, “ideologies”, or “cosmoses” [16] to consensually develop a reporting template. Therefore, we recommend interested stakeholders of the PLIRT to use Table 2 as a template for adequately reporting the main characteristics of PL interventions.

The present study has three major limitations. First, the maximum number of participants and the a priori defined criteria to cover a broad spectrum of perspectives (see Sect. 2.2) made it necessary to undertake a deliberate selection of experts. Despite the awareness of actor networks [16], the composition of the group may have been influenced by existing collaborations reflecting similar assumptions and mindsets of PL. Second, the selected methodology concentrated on academic experts. Although this is in line with methodological guidelines [55] and the fact that this work aimed at strengthening the theoretical underpinnings of PL interventions, the PLIRT development may have additionally benefitted from practitioner involvement. Lastly, Africa and South America were not represented in the expert round. Although this circumstance basically reflects the current situation with regard to publications within the field of PL and PL interventions, in particular [46, 102], important cultural perspectives were missing in the expert panel.

## Conclusion

Existing interventions adhering to the PL approach often lack sound theoretical foundation and basic information for the delivery. The Physical Literacy Interventions Reporting Template (PLIRT) constitutes an attempt to enable more coherent interpretation and synthesis of findings in the context of PL interventions. The PLIRT with its 14 (or 16, respectively) items serves as a minimum orientation for stakeholders of PL interventions. Developers and authors can benefit from the template in developing the PL intervention and in structuring their corresponding reports; finally, editors as well as reviewers can better interpret the completeness of intervention description to improve the reader’s understanding of published material.

## Supplementary Information

**Additional file 1: Table 1. **Items of the guidance for the development of research reporting guidelines [51]. **Table 2. **Comparison of different definitions and their included domains.

## Data Availability

Not applicable.

## Abbreviations

PL: Physical Literacy
PLIRT: Physical Literacy Interventions Reporting Template
PA: Physical Activity
IPLA: International Physical Literacy Association
TIDieR: Template for intervention description and replication
CERT: Consensus on exercise reporting template
