# Supplementary material for: Development, explanation, and presentation of the Physical Literacy Interventions Reporting Template (PLIRT)
Source: Int J Behav Nutr Phys Act. 2023 Feb 18;20:21. doi: 10.1186/s12966-023-01423-3 (PMC9938627; doi:10.1186/s12966-023-01423-3)
Supplement: Supplementary file 1 — Additional file 1: Table 1. Items of the guidance for the development of research reporting guidelines [51]. Table 2. Comparison of different definitions and their included domains. [file 12966_2023_1423_MOESM1_ESM.docx]

**Supplementary Tables**

**Table 1** Items of the guidance for the development of research reporting guidelines [51].

| **Item Nr.** | **Description** | **Comment / Location of Information in the Study** |
| --- | --- | --- |
| ***INITIAL STEPS*** | | |
| 1 | Identify the need for a guidance | Need is explained in the background section |
| 1.1 | Develop new guidance |  |
| 2 | Review the literature | A systematic review was the starting point for this initiative, with the results guiding the development process (see figure 1 as well as sections 1.2, 2.2, and 2.4) |
| 2.1 | Identify previous relevant guidance | A search was conducted; the TIDieR checklist and the CERT are other, related reporting frameworks and should be combined with the PLIRT (see figure 1, sections 1.2 and 2.4, as well as item 10 in the results section) |
| 2.2 | Seek relevant evidence on the quality of reporting in published research activities | A systematic review was the starting point for this initiative, with the results guiding the development process (see sections 1.2, 2.2, and 2.4) |
| 2.3 | Identify key information related to the potential sources of bias in relevant studies | This information can also be retrieved from the initial systematic review which used the Theory Coding Scheme and therefore revealed gaps in links between theory and content of interventions (see sections 1.2, 2.2, and 2.4) |
| 3 | Obtain funding for the guideline initiative | The present project was internally funded and defined as part of a post-doctoral employment |
| ***PRE-MEETING ACTIVITIES*** | | |
| 4 | Identify participants | Extensive explanation in section 2.2 with clear criteria to consider participants as experts |
| 5 | Conduct a delphi exercise | Description in figure 1 as well as sections 2.1, 2.2, and 2.3 |
| 6 | Generate a list of items for consideration at the face-to-face meeting | Description in sections 2.2 and 2.3 |
| 7 | Prepare for the meeting | See figure 1 as well as sections 2.2 and 2.3 |
| 7.1 | Decide size and duration of the face-to-face-meeting | See sections 2.2 and 2.3 |
| 7.2 | Develop meeting logistics | See sections 2.2 and 2.3 |
| 7.3 | Develop meeting agenda | See sections 2.2 and 2.3 |
| ***CONSENSUS MEETING ITSELF*** | | |
| 8 | Present and discuss results of pre-meeting activities and relevant evidence | See section 2.3 |
| 8.1 | Discuss the rationale for including items in the checklist | The discussion was part of the synchronous meetings (section 2.3); individual and anonymous voting was ensured (section 2.3); the final rationale and explanation for each item is part of the result section |
| 8.2 | Discuss the development of a flow diagram | It was decided to not include a flow diagram but a theoretical model aiming to improve the infusion of PL theory into the intervention at all stages of the development process (see figure 2) |
| 8.3 | Discuss strategy for producing documents; identify who will be involved in which activities; discuss authorship | The present article is the result of this process; all authors have substantially contributed to the publication and provided consent to authorship (see declarations section); except of first and senior author, alphabetical order of authorships |
| 8.4 | Discuss knowledge translation strategy | This article and part of planned activities after this publication |
| ***POST-MEETING ACTIVITIES*** | | |
| 9 | Develop the guidance statement | The development process has provided several iterations (see figure 1 as well as sections 2.1 and 2.3); all items are presented with explanations in the results section |
| 9.1 | Pilot-testing | Pilot-testing was not part of this article (see transparent declaration in section 2.1 and the publication strategy item 11 here) |
| 10 | Development an explanatory document | This article not only serves to describe the methodological background for the construction of PLIRT but also separately lists and explains each item in the results section |
| 11 | Develop a publication strategy | The present article represents the first publication |
| ***POST-PUBLICATION ACTIVITIES*** | | |
| 12 | Seek and Deal with Feedback and Criticism | Part of planned activities after this publication |
| 13 | Encourage Guideline Endorsement | Part of planned activities after this publication |
| 14 | Support Adherence to the Guideline | Part of planned activities after this publication |
| 15 | Evaluate the Impact of the Reporting Guidance | Part of planned activities after this publication |
| 16 | Develop Website | Part of planned activities after this publication |
| 17 | Translate Guideline | Part of planned activities after this publication |
| 18 | Update Guideline | Part of planned activities after this publication |

Abbreviations: PL = Physical literacy; TIDieR = template for intervention description and replication; CERT = consensus on exercise reporting template.

*Table 2.* Comparison of different definitions and their included domains.

| **Country** | **Main  References** | **Inclusion of Domains** | | | | |
| --- | --- | --- | --- | --- | --- | --- |
|  |  | **Physical (“Physical Competence”)** | **Cognitive (“Knowledge and Understanding”)** | **Affective/Emotional/ Psychological (“Motivation and Confidence”)** | **Social** | **Spiritual** |
| Australia | 13, 14 | x | x | x | x |  |
| Canada | 63 | x | x | x |  |  |
| China | 64 | x | x | x |  |  |
| New Zealand | 15 | x | x | x |  | x |
| Wales | 65 | x | x | x |  |  |
| IPLA | 9 | x | x | x |  |  |

Note: It cannot be excluded that longer conceptual texts contain elements that may implicate the relevance of another aspect (e.g., “lots of opportunities” may be interpreted as raising the question about a potential environmental factor as part of PL); IPLA = International Physical Literacy Association.
